# Predictors of Cognitive Decline in the Early Stages of Parkinson's Disease: A Brief Cognitive Assessment Longitudinal Study

**DOI:** 10.1155/2013/912037

**Published:** 2013-11-05

**Authors:** Paulo Bugalho, Miguel Viana-Baptista

**Affiliations:** ^1^Neurology Department, Hospital de Egas Moniz, CHLO, Rua da Junqueira 126, 1349-019 Lisbon, Portugal; ^2^CEDOC and Neurology Department, Faculdade de Ciências Médicas (FCM), Universidade Nova de Lisboa (UNL), Campo dos Mártires da Pátria, 130, 1169-056 Lisbon, Portugal

## Abstract

Our objectives were to perform a longitudinal assessment of mental status in early stage Parkinson's disease (PD) patients, with brief neuropsychological tests, in order to find predictive factors for cognitive decline. Sixty-one, early stage, and nondemented patients were assessed twice, over a 2-year interval, with a global cognitive test (mini-mental state examination (MMSE)) and a frontal function test (frontal assessment battery (FAB)) and motor function scales. Dementia and hallucinations were diagnosed according to the DSM-IV criteria. Cognitive function scores did not decrease significantly, except for FAB lexical fluency score. Four patients presented with dementia at followup. The MMSE score below cut-off, worse gait dysfunction, the nontremor motor subtype, and hallucinations were significantly related to dementia. Rigidity and speech dysfunction were related to dementia and a decrease in FAB scores. We can conclude that decline in the MMSE and FAB scores is small and heterogeneous in the early stages of PD. Scores below cut-off in the MMSE could be helpful to predict dementia. Nontremor motor deficits could be predictive factors for frontal cognitive decline and dementia.

## 1. Introduction

Parkinson's disease is a movement disorder, defined by a combination of tremor, rigidity, bradykinesia, and gait disturbances [1]. Lately, a constellation of nonmotor symptoms has also been described [2]. Cognitive dysfunction, which can ultimately lead to dementia in a great number of cases [3], is a cause of great incapacity in PD. As therapeutic alternatives develop [4], the need for an early detection of cognitive deficits and for accurate prediction of cognitive outcome increases. Brief cognitive tests could be useful for a rapid screening of patients at higher risk for cognitive decline. They also could be of use for following cognitive decline and predicting cognitive outcome. Noncognitive symptoms at the baseline, like motor dysfunction severity, or specific motor symptoms could also be useful as cognitive outcome predictors. Several studies have defined significant clinical heterogeneity at disease onset [5, 6] which could determine prognosis. In previous work, we found that early stage, nondemented PD patients presented with significantly lower scores in the frontal assessment battery (FAB) and the mini-mental state examination (MMSE), when compared to non-PD aged controls, and that MMSE scores were related to nontremor motor scores [7]. In the present study, our objectives were to perform a longitudinal analysis of this cohort, in order to assess the relation between motor and cognitive performance at baseline and cognitive dysfunction progression.

## 2. Methods

Seventy-five early stage PD patients, diagnosed according to validated criteria [1], were consecutively recruited from Hospital Egas Moniz Neurology Department's outpatient clinic. Exclusion criteria were the existence of relevant psychiatric, medical or other neurological diseases. The early stage PD was defined as disease duration (time in years from appearance of first motor symptoms to study first assessment) up to 5 years and the Hoehn and Yahr [8] (HY) stage from 1 to 2.5, included, at baseline.

Patients were assessed twice, with a two-year interval (*t*_0_ and *t*_1_), with the same instruments and by the same observer. 

### 2.1. Motor Assessment

Patients were assessed with the unified Parkinson's disease rating scale (UPDRS) parts II and III [9], after receiving their usual medication and while on *on* state. Separated scores were derived for tremor, rigidity, bradykinesia, speech, and gait/postural stability symptoms, from items 20 and 21, 22, 23 to 27, 18, and 29 to 30, respectively. Patients were split into tremor, intermediate, and postural instability and gait difficulty (PIGD) predominant motor groups, according to the classification system proposed by Jankovic and coworkers [10]. For statistical purposes, this variable was dichotomized in tremor and nontremor (PIGD + intermediate). Dopaminergic treatment was calculated as L-dopa equivalent doses (DED) [11].

### 2.2. Cognitive Function Assessment

#### 2.2.1. MMSE

 Global cognitive function was evaluated with the MMSE [12]. The MMSE is a widely used bed-side test, which assesses orientation, verbal memory, language, attention/calculation, and visuoconstructive abilities. While some studies have challenged. MMSE efficacy as a screening instrument in Parkinson's disease, because it lacks specific tests for executive function assessment [13–15], others have found the MMSE useful to detect cognitive deterioration in the early stage PD [16] and also a significant correlation between the MMSE scores and cortical hypometabolism [17], motor symptoms [18–20], and neuropathological status [21]. The MMSE has been recommended by the movement disorder society task force for level I testing, to assess *PD* associated with a decreased global cognitive efficiency and also for detecting impairment in more than one cognitive domain [22] and has been previously used to characterize pd dementia in clinical trials [4, 23]. 

#### 2.2.2. FAB

Frontal function was assessed with the FAB [24]. The frontal assessment battery (FAB) is a rapid screening battery (taking approximately ten minutes), which evaluates several frontal function domains (conceptualization, mental flexibility, motor programming, sensitivity to interference, inhibitory control, and environmental autonomy), and it has been validated for PD [25–27]. Studies have shown high correlation with classical frontal neuropsychological tests [24], significant differences between patients and controls [25, 26], and correlation between the FAB performance and perfusion in the medial and dorsolateral frontal cortex [27]. 

Portuguese validated versions of the tests were used [28, 29]. We determined the number of patients with global (MMSE) and frontal (FAB) cognitive dysfunctions, using published cut-off scores for the Portuguese versions of the tests [28, 29]. Cut-off scores for the MMSE were the following: 22 (0 to 2 years of schooling); 24 (3 to 6 years of schooling); 27 (7 or more years of schooling) (scores below the cut-off scores were considered as signifying global cognitive dysfunction). Patients were considered to have frontal dysfunction if they scored one standard deviation below healthy controls mean score of the same age group, as presented in the validation study of the Portuguese version of the test. As reported by the authors, the normative study involved 122 control subjects (68 women and 54 men) who varied widely in age and education. They were from various regions of Portugal, from urban and nonurban areas. None of the participants had any conditions that could affect the mental state, as assessed by an individual clinical interview.

 Given that the instruments were applied by the same observer in two different time points, it is important to verify the test-retest variability of the tests and the their intrarater reliability to make sure that the changes we observe are real and not influenced by the variability of the test itself. The validity and reliability of the FAB were confirmed by several studies [30, 31]. Test-retest reliability (intrarater reliability) was found to be adequate. Siderowf et al. [32] have validated the UPDRS and found high intrarater reliability for the total score of part three and also for the tremor, bradykinesia and rigidity subscales. The construct validity of these subscales had been previously verified by McDermott et al. [33]. Validation studies have also confirmed the intrarater and test-retest reliability of the mini-mental state examination [34]. The study by Aarsland and collaborators [35], performed on the PD patients, suggests that the MMSE is valid to measure the change in cognitive state for periods of at least one year.

 Dementia and the presence of hallucinations were diagnosed according to the DSM-IV-R criteria [36]. Hallucinations were defined as a sensory perception that has the compelling sense of reality of a true perception but that occurs without external stimulation of the relevant sensory organ. Dementia was defined, after exclusion of other causes, as the progressive development of cognitive deficits causing at least two of the following: memory impairment, language disturbance, apraxia, agnosia, and disturbance in executive functioning. The cognitive deficits cause a significant impairment in social or occupational functioning and represent a significant decline from a previous level of functioning. We used the incapacity to independently manage the PD medication as criteria for the significant impact of cognitive dysfunction in daily live activities, as suggested in [22]. 

### 2.3. Data Analysis

The incidence of dementia was calculated by dividing new cases of dementia by the number of person-years at risk throughout the observation period. Because it is not possible to know the precise time a subject became demented, it was assumed to occur in the midpoint of the period of observation.

 In a first analysis, we compared the differences in the FAB and the MMSE total and partial scores between *t*_0_ and *t*_1_, using paired sample *t*-tests or the Wilcoxon tests (depending on the distribution of the samples). Variations in FAB and MMSE scores were also calculated for each patient, as a measure of change: score at *t*_1_ − score at *t*_0_. Paired proportions (proportion of patients with global cognitive at *t*_0_ versus at *t*_1_, proportion of patients with frontal dysfunction at *t*_0_ versus at *t*_1_) were compared by means of McNemar's tests.

 In a second analysis, we studied the relation between cognitive tests scores and clinical variables at *t*_0_ and the presence of cognitive decline at *t*_1_. Predictor variables (independent variables) were UPDRS total score, bradykinesia, rigidity, tremor, speech, gait-posture scores, Hoehn and Yahr scores, age, age of onset, diration of disease, education, gender, DED, global cognitive dysfunction, frontal cognitive dysfunction and presence of hallucinations. Outcome variables (dependent variables) were dementia at *t*_1_, change in the MMSE, and change in the FAB. To test the relation between predictor and outcome variables, the former were dichotomized at the median. Fisher's exact test was used to test the relation between predictor variables and dementia, and Mann-Whitney tests were used to test the relation between predictor variables and the MMSE and FAB change. Multivariate analysis was not performed, either because there were few positive cases (dementia) or because few variables were found as significant predictors (FAB and MMSE change). 

 In a third analysis, we aimed at evaluating clinical heterogeneity in the early stages of Parkinson's disease and its influence on cognitive decline. A two-step cluster analysis was performed, in which we entered all predictor variables. Differences between the clusters thus formed were tested by means of two-samples *t*-tests or chi-square tests. 

## 3. Ethics

Patients signed informed consent forms, and the ethics committee of the institution approved the protocol.

## 4. Results

Of the 75 patients assessed at *t*_0_, 61 were reassessed at *t*_1_. Fourteen were not reassessed: 2 died from causes unrelated to PD; 1 refused reassessment; diagnosis changed in 4, which at follow-up were found not to have PD; 7 patients were out of contact and were lost for follow-up. Dropouts showed a tendency for older age and older age of onset. Frontal dysfunction was significantly more frequent in this group. There were no other significant differences in demographic, motor, or cognitive variables between patients lost for follow-up and the rest of the cohort (Table 1).

At *t*_0_, no patient was demented against 4 at *t*_1_ (incidence of 33.9/1000 per year). The patients with global cognitive impairment were the same at *t*_0_ and *t*_1_ (*n* = 6). The MMSE scores decreased in 23 patients (mean change −1.91 ± 1.20, range from −1 to −5), increased in 21 patients (mean change 1.81 ± 0.98, range from 1 to 5), and did not change in 17 patients. The frequency of frontal cognitive dysfunction increased from 26 to 32, but the difference did not reach significance (*P* = 0.201). The FAB scores decreased in 29 patients (mean change −2.04 ± 1.37, range from −1 to −5), increased in 23 patients (mean change 2.87 ± 1.74, range from 1 to 7), and did not change in 9 patients.

There were no significant variations in the MMSE total and subscores (Table 2).

Changes in the FAB were significant for lexical fluency task (lower at *t*_1_) (Table 3). 

Table 4 shows the relation between predictive variables at *t*_0_ and the occurrence of dementia. Higher speech, rigidity, gait/posture scores, and the presence of hallucinations and of the nontremor phenotype were significantly related to dementia. 

Table 5 shows the relation between variables at *t*_0_ and the changes in the MMSE and FAB scores. Frontal cognitive dysfunction and higher education were significantly related to the decreases in the MMSE scores. As chi-square analysis revealed that these two variables were significantly correlated (frontal dysfunction being more frequent in patients with higher education: 57.1% versus 23.1%, *P* = 0.010), they were included as predictor variables in a linear regression analysis model, with the MMSE variation as an outcome variable. This showed that only frontal dysfunction had a significant influence in the MMSE variation (*b* = 1.37,  *P* = 0.003). 

Higher rigidity and speech scores were significantly related to the decreases in the FAB scores (Table 5). 

Cluster analysis yielded two clusters. Compared to patients in cluster 2 (*n* = 35), cluster 1 (*n* = 26) patients presented with significantly higher HY stage (*P* < 0.00001), UPDRS total score (*P* < 0.00001) dysarthria (*P* < 0.00001), tremor (*P* = 0.019), rigidity (*P* = 0.004), bradykinesia (*P* < 0.00001), gait and postural instability (*P* < 0.00001) scores, and a significantly higher prevalence of the nontremor phenotype (*P* < 0.00001). Patients with hallucinations were all included in cluster 1 (*P* = 0.029). Patients in cluster 1 showed a more significant decrease in the FAB scores (*P* = 0.040). Patients who subsequently developed dementia were all included in cluster 1 (*P* = 0.029).

## 5. Discussion

### 5.1. Variation of the MMSE and FAB Scores over Time

Variation in the MMSE mean scores from *t*_0_ to *t*_1_ was nonsignificant. Mean change was lower than in previous studies [35, 37, 38] and closer to that of Gago et al. [20] and of Williams-Gray et al.'s [39] studies, who also evaluated early stage patients. A slower rate of decline in the first stages of disease is in accordance with Aarsland et al. study [38] that has shown a nonlinear pattern of decline in the MMSE, with an inflection point at about 11 years duration, beyond which decline was steeper, reaching a mean of 2.8 points annually. More recently, Lessig et al. [40] also found that the MMSE variation was more pronounced after the first 10 years of disease. A third of the patients presented a decline in the MMSE total scores. Improvement in other patients, however, compensated these changes, resulting in almost null mean variation over time. Although the short time interval between evaluations may have promoted a learning effect, contributing to an improvement on follow-up assessment, the different pattern of cognitive change could argue in favor of a heterogeneous progression of neuropsychological deficits in the early stages of PD. It also suggests that the MMSE may not be indicated for detecting cognitive change at these stages of disease, which could require more demanding measures of cognitive dysfunction. Frontal dysfunction progression was also heterogeneous. Significant variations occurred only in relation with lexical fluency, suggesting that this could be fit to measure cognitive changes at these stages of disease. Significant progression in lexical fluency deficits was also found by Azuma et al. [41] and Levy et al. [42]. 

### 5.2. Predictors of Dementia

Only four patients presented dementia at follow-up (annual incidence 33.9/1000). Incidence was lower than in most studies, in which this figure ranged from 30 to 107/1000 [38, 43, 44]. This could be due to the specific nature of our cohort, which was constituted only by early stage disease patients and also to the relatively short follow-up time. Previous work has shown that dementia incidence increases as the disease progresses, and the lower values are found in studies performed in early stage patients [45]. This is also in accordance with the wide believed notion that dementia is a late finding in PD. 

Nontremor motor scores, nontremor motor phenotype, the presence of hallucinations, and scores below cut-off in the MMSE were related to dementia at follow-up. Significant relation between hallucinations and cognitive dysfunction in PD has been found in several studies [46, 47]. Nondemented PD patients with hallucinations show significant atrophy in frontal and occipitotemporal cortical regions whose progression was linked to cognitive deterioration in longitudinal studies [47], meaning that hallucinations could be a sign of early neuropathological changes leading to dementia in the long run. Nontremor motor phenotype was significantly more frequent in patients that subsequently developed dementia, as also found in previous longitudinal studies [48]. Axial motor dysfunction, like gait and speech disturbances, responds poorly to dopaminergic treatment, and some authors have proposed that cholinergic dysfunction could contribute to these symptoms. PD dementia has also been associated with cholinergic rather than dopaminergic deficits, and some have suggested that dementia and gait dysfunction could have the same physiopathological basis, originating from cholinergic deficits caused by pedunculo-pontine and of nucleus basalis the Meynert degeneration [42]. 

Previous work has suggested that the MMSE could be less sensitive to mild cognitive impairment, when compared to frontal oriented tests, like the Montreal cognitive assessment scale [13–15], although it is better for tracking cognitive changes over time in PD [32]. Our data suggests that the MMSE, and particularly the cut-off values, determined in the validation study of the Portuguese version of the test, could be useful for predicting PD dementia in early stage patients. The presence of cognitive deficits in patients that eventually develop a state of full-blown dementia suggests that cognitive dysfunction is a progressive disorder in PD, and that dementia, as happens with Alzheimer's disease, is preceded by a state of subtler neuropsychological dysfunction, compatible with the notion of mild cognitive impairment. 

Contrary to the MMSE scores, the FAB scores were not useful in predicting dementia. This could be related to the nature of cognitive deficits in PD-related dementia. Previous investigation has shown a significant relation between the progression to dementia and the superimposition of non-frontal deficits in patients with frontal deterioration [49]. In this way, a global, non-frontal test could be more helpful to predict dementia than a frontal-oriented one. 

### 5.3. Predictors of Global and Frontal Cognitive Decline

No variable was predictive of a decline in the MMSE, except for the presence of frontal dysfunction. Although our data does not allow for a straightforward explanation for these findings, we could hypothesize that, frontal dysfunction being the first step in PD-related cognitive deterioration, as mentioned above, it could act as a predictor in some patients of widespread decline of cognitive function. On the other hand, some studies [24, 25] have found significant correlations between FAB and MMSE, which could have biased our results. 

Similar to dementia, frontal dysfunction was predicted by nontremor motor symptoms, although the pattern was slightly different. In particular, patients with higher gait and postural dysfunction scores at baseline did not present a greater decline in the FAB scores. As previously discussed, gait dysfunction seems to be mostly correlated to cholinergic rather than to dopaminergic deficits. Other authors found an association between executive dysfunction and dopamine responsive symptoms, while cortical posterior deficits were associated with dopamine resistant symptoms [49]. Williams-Gray and coworkers [39] have found that the frontal type deficits at the early stages of disease are not related to dementia at follow-up but to COMT gene mutations, that is, to dopamine dysfunction. They hypothesized that there could be a dissociation between frontal/dopaminergic related dysfunction, bearing no relation with dementia at follow-up, and MAPT/ageing/posterior type of cognitive deficits, which could be predictive of dementia. The relation between PD-related dementia and cholinergic dysfunction could explain the improvement of cognitive function in PD patients treated with cholinesterase inhibitors 2. 

### 5.4. Cluster Analysis

 Cluster analysis showed two groups that differed regarding the presence of hallucinations and the severity of nontremor motor symptoms. The cluster with worse nontremor symptoms and hallucinations showed a steeper progression of frontal dysfunction and a higher prevalence of dementia, which is in accordance with results from the previous analysis. Comparison with other studies is difficult, because cluster analysis depends on the variables that are included. However, Erro and collaborators [5] did find a cluster in which worse axial motor symptoms are correlated with worse outcome. Lewis et al. also found a group with worse nontremor motor symptoms and significant levels of cognitive impairment [6].

The present study has several limitations, which should be taken into account when discussing the data. Because we assessed an early stage cohort, and the follow-up time was relatively short, the ratio of dementia conversation was very low. This prevents multivariate analysis procedures, weakens the statistical analysis, and precludes definite conclusions regarding the predictive factors for dementia in this group. Patients were recruited from a tertiary centre outpatient clinic, and the sample was small, precluding the generalization of results to an epidemiological level—it does, however, mirror the clinical experience of a Portuguese neurological center and could be informative in that context. 

In conclusion, our data suggests that nontremor motor deficits are predictive factors for dementia and frontal type decline in the early stages of Parkinson's disease. They also suggest that mini-mental state examination cut-off scores could be useful for predicting dementia in these stages. 

## Figures and Tables

**Table 1:** Comparison between patients who remained in observation and those lost for follow-up.

	Remained in observation (*n* = 61)	Lost for follow-up (*n* = 14)	*P*
Gender (m)	28	4	0.370
Tremor subtype	34	7	0.977
Hallucinations (*n* = 4)	4	1	1.000
FAB < cut-off (*n* = 17)	17	9	0.014*
MMSE < cut-off (*n* = 6)	4	2	0.638
Age	71.9 ± 7.53	75.4 ± 4.55	0.099
Age of onset	69.1 ± 7.76	72.7 ± 4.75	0.098
Duration	2.8 ± 1.40	2.7 ± 1.27	0.891
Education	4.4 ± 4.22	4.1 ± 3.46	0.858
HY stage	1.8 ± 0.59	1.8 ± 0.54	0.845
DED	390.0 ± 355.80	487.5 ± 695.49	0.453
UPDRS total	18.6 ± 11.05	15.5 ± 13.92	0.374
Dysarthria	0.6 ± 0.75	0.6 ± 0.75	0.987
Tremor	4.1 ± 3.71	4.1 ± 4.37	0.946
Rigidity	2.3 ± 3.07	1.7 ± 1.20	0.551
Bradykinesia	7.4 ± 6.19	5.8 ± 6.73	0.377
Gait/posture	1.4 ± 1.14	1.6 ± 1.28	0.609

Values are mean ± standard deviation, except for categorical variables.

**Table 2:** Mini-mental state examination scores' variation.

MMSE scores	*t*_0_	*t*_1_	Mean variation	*P*
Total score	27.20 (2.544)	27.11 (2.745)	−0.098 (4%)	0.678
Orientation	9.59 (0.844)	9.48 (0.924)	−0.115 (1%)	0.289
Registration	3.00	3.00	0	
Attention and calculation	4.00 (1.252)	4.07 (1.276)	0.066 (2%)	0.636
Recall	2.31 (0.720)	2.39 (0.737)	0.082 (4%)	0.450
Language	7.62 (0.522)	7.67 (0.598)	0.492 (1%)	0.496
Visuoconstructive ability	0.66 (0.479)	0.54 (0.502)	−0.115 (17%)	0.090

Values are mean standard deviation or mean variation (percent variation). Negative value means decrease from *t*_0_ to *t*_1_. *P* value is significance for paired sample student's *t*-tests. Registration scores remained constant throughout the study.

**Table 3:** Frontal assessment battery scores' variation.

FAB scores	*t*_0_	*t*_1_	Mean variation	*P*
Total	11.20 (2.971)	11.30 (3.309)	0.115 (1%)	0.738
Similarities	0.90 (0.810)	0.87 (0.826)	−0.033 (4%)	0.766
Lexical fluency	1.93 (0.873)	1.72 (0.968)	−0.213 (11%)	0.022*
Motor series	2.15 (0.946)	2.25 (0.925)	0.0984 (4%)	0.451
Conflicting instructions	1.77 (1.131)	1.92 (1.187)	0.1475 (8%)	0.268
Go-No Go	1.51 (0.868)	1.56 (1.009)	0.0492 (3%)	0.717
Prehension behaviour	3	3	0	

Values are mean standard deviation or mean variation (percent variation). Negative value means decrease from *t*_0_ to *t*_1_. *P* value is significance for paired sample student's *t*-tests. Prehension behaviour scores remained constant throughout the study. *P < 0.05.

**Table 4:** Baseline comparisons between the PD patients with and without dementia.

	Dementia		
	Yes (*n* = 4)	No (*n* = 57)	*P*
Gender (m)	2 (50.0)	26 (45.6)	1.000
Tremor subtype	0 (0)	34 (59.6)	0.034*
Hallucinations	2 (50.0)	2 (3.5)	0.019*
FAB	3 (75.0)	14 (40.0)	0.303
MMSE	3 (75.0)	3 (5.3)	0.002**
Age > 72	4 (100)	28 (49.1)	0.114
Age of onset > 69	4 (100)	29 (50.9)	0.118
Duration > 2	2 (50)	29 (50.9)	1.000
Education > 3	2 (50)	33 (57.9)	1.000
HY stage > 1	4 (100)	36 (63.2)	0.289
DED > 395	4 (100)	29 (50.9)	0.118
UPDRS total > 16	4 (100)	29 (50.9)	0.118
Speech	4 (100)	25 (43.9)	0.046*
Tremor > 4	2 (50)	17 (29.8)	0.582
Rigidity > 1	4 (100)	24 (42.1)	0.039*
Bradykinesia	4 (100)	26 (45.6)	0.053
Gait/posture > 1	4 (100)	25 (43.9)	0.046*

Values are number of patients (percentage). *P* value is significance for chi-square statistics. *P < 0.05, **P < = 0.01.

**Table 5:** Baseline comparisons regarding the MMSE and FAB changes from *t*_0_ to *t*_1_.

Predictor variables (*t*_0_)	MMSE change		FAB change	
	Mean variation	*P*	Mean variation	*P*
Gender				
Male	−0.21 (1.75)	0.871	−0.21 (3.05)	0.244
Female	0.69 (1.93)		0.39 (2.30)	
Motor subtype				
Tremor	0.00 (1.46)	0.842	0.50 (2.34)	0.186
Nontremor	−0.22 (2.26)		−0.30 (2.98)	
Hallucinations				
Yes	0.00 (4.40)	0.965	0.50 (1.43)	0.680
No	−0.10 (1.67)		0.12 (2.54)	
Frontal dysfunction				
Yes	−0.88 (2.27)	0.002**	0.46 (2.85)	0.474
No	0.49 (1.17)		−0.09 (2.51)	
Global cognitive dysfunction				
Yes	0.00 (3.69)	0.980	−0.83 (2.04)	0.207
No	−0.11 (1.58)		0.25 (2.70)	
Age				
72	0.09 (2.27)	0.239	0.41 (2.84)	0.296
≤72	−0.31 (1.23)		−0.21 (2.47)	
Age of onset				
69	0.18 (2.21)	0.072	0.52 (2.84)	0.247
≤69	−0.43 (1.23)		−0.28 (2.39)	
Duration				
2	−0.58 (1.88)	0.080	−0.48 (2.74)	0.077
≤2	0.40 (1.69)		0.80 (2.43)	
Education				
3	−0.46 (1.62)	0.048*	−0.03 (2.57)	0.723
≤3	0.38 (2.04)		0.31 (2.83)	
HY stage				
1	−0.95 (1.76)	0.914	−0.28 (2.57)	0.051
1	−0.10 (1.91)		0.95 (2.67)	
DED				
395	0.04 (1.40)	0.819	0.68 (2.39)	0.101
≤395	−0.21 (2.16)		−0.36 (2.83)	
UPDRS total				
>16	−0.03 (1.31)	0.507	−0.15 (2.86)	0.160
≤16	−0.18 (1.31)		0.43 (2.42)	
Speech				
>0	−0.10 (2.19)	0.774	−0.55 (2.96)	0.022*
0	−0.10 (1.49)		0.78 (2.20)	
Tremor				
>4	−0.37 (2.57)	0.471	0.37 (2.64)	0.671
≤4	0.02 (1.42)		0.05 (2.68)	
Rigidity				
>1	−0.21 (2.13)	0.586	−0.54 (2.49)	0.020*
≤1	0.00 (0.16)		0.73 (2.68)	
Bradykinesia				
>7	−0.13 (2.19)	0.978	−0.03 (2.66)	0.350
≤7	−0.06 (1.46)		0.32 (2.68)	
Gait/posture				
>1	−0.25 (1.70)	0.471	−0.28 (3.03)	0.111
≤1	0.07 (2.00)		0.53 (2.23)	

Values are mean standard deviation. *P* value is significance for Mann-Whitney tests.
